# Citizens’ digital footprints to support health promotion at the local level—PUHTI study, Finland

**DOI:** 10.1093/eurpub/ckae053

**Published:** 2024-04-04

**Authors:** Katri Kilpeläinen, Timo Ståhl, Tiina Ylöstalo, Teemu Keski-Kuha, Riku Nyrhinen, Päivikki Koponen, Mika Gissler

**Affiliations:** Department of Public Health and Welfare, Finnish Institute for Health and Welfare, Helsinki, Finland; Department of Public Health and Welfare, Finnish Institute for Health and Welfare, Helsinki, Finland; Department of Knowledge Brokers, Finnish Institute for Health and Welfare, Helsinki, Finland; Department of Public Health and Welfare, Finnish Institute for Health and Welfare, Helsinki, Finland; Department of Public Health and Welfare, Finnish Institute for Health and Welfare, Helsinki, Finland; Department of Public Health and Welfare, Finnish Institute for Health and Welfare, Helsinki, Finland; Department of Knowledge Brokers, Finnish Institute for Health and Welfare, Helsinki, Finland; Region Stockholm, Academic Primary Health Care Centre, Stockholm, Sweden; Department of Molecular Medicine and Surgery, Karolinska Institutet, Stockholm, Sweden

## Abstract

**Background:**

We aimed to explore to the possibilities of utilizing automatically accumulating data on health—owned for example by local companies and non-governmental organizations—to complement traditional health data sources in health promotion work at the local level.

**Methods:**

Data for the PUHTI study consisted of postal code level information on sport license holders, drug purchase and sales advertisements in a TOR online underground marketplace, and grocery sales in Tampere. Additionally, open population register data were utilized. An interactive reporting tool was prepared to show the well-being profile for each postal code area. Feedback from the tool’s end-users was collected in interviews.

**Results:**

The study showed that buying unhealthy food and alcohol, selling or buying drugs, and participating in organized sport activities differed by postal code areas according to its socioeconomic profile in the city of Tampere. The health and well-being planners and managers of Tampere found that the new type of data brought added value for the health promotion work at the local level. They perceived the interactive reporting tool as a good tool for planning, managing, allocating resources and preparing forecasts.

**Conclusions:**

Traditional health data collection methods—administrative registers and health surveys—are the cornerstone of local health promotion work. Digital footprints, including data accumulated about people’s everyday lives outside the health service system, can provide additional information on health behaviour for various population groups. Combining new sources with traditional health data opens a new perspective for health promotion work at local and regional levels.

## Introduction

The main goals of democratic societies are to promote the well-being of residents and to reduce health inequalities. Despite the positive development in general, health and well-being continue to accumulate unfairly between population groups.^1–4^

The major public health challenges in the most European countries include overweight, type 2 diabetes, cardiovascular diseases, preventable cancers and mental health disorders. These are all associated with lifestyle factors, such as unhealthy diet, low physical activity, smoking, alcohol abuse and drug use. These risk factors tend to accumulate for some areas and population groups, like those with the shortest education or the lowest income, which increases health inequalities.^1^^,^^4–6^

Up-to-date information on health and well-being of the population can be used in recognizing the risk groups, and for targeting the health promotion interventions effectively at the local level.^1^^,^^7–10^ However, the collection of health information on the various socio-economic groups is time-consuming and expensive using traditional data collection methods—such as health surveys and administrative registers. Such information might not even be possible to get or publish for the smallest areas due to methodological and privacy issues. In addition, health survey response rates have been declining for the past 20 years, especially among young people.^11–14^

There is a growing interest in finding and evaluating new ways to collect automatically accumulating data on health behaviour to complement traditional health data sources to be utilized at the local management. So far new types of data, e.g. from private companies and non-governmental organizations have provided added value mostly for business, but its opportunities also for public health research have been noticed.^10^ These data sources might have the potential to complete the traditional information due to the data size, coverage, objectivity, low costs and time trends for the data collection, even though the data are not collected with public health as an objective.^5–7^^,^^10^^,^^15–24^

The aim of this study is to explore the possibilities of utilizing citizen’s digital footprints—data accumulated about the people’s everyday lives—to support municipal experts and decision-makers to plan, carry out and lead the health promotion activities at the local level. We will describe the development process of a new interactive reporting tool based on new data sources in Finland and feedback from the end-users in municipal health promotion.

## Methods

### PUHTI pilot study 2019–2020

In 2016, the Finnish Digitalization programme of the Ministry of Social Affairs and Health identified the need to collect and utilize health-related information, which is accumulated about the people’s everyday lives outside the health service system. The PUHTI pilot study (later PUHTI) was one of the implementation actions under this programme. It was coordinated and funded by the Finnish Institute for Health and Welfare (THL).^25^

PUHTI was carried out in September 2019 to December 2020 in Tampere, which was the third biggest city in Finland with 241 009 inhabitants in 2020.^26^ The study included collaboration with private companies and non-governmental organizations, data mining, data collection, data analysing and developing an interactive reporting tool that showed the health profiles for each postal code area in Tampere.^25^

PUHTI aimed to explore data, that (a) was considered as describing key factors affecting the health and well-being of young people, (b) accumulated about people’s everyday lives outside the health service system and (c) could be influenced by health programmes and interventions.

First, the willingness for collaboration was investigated from various private companies and organizations, because their role as data owners was central to the study. As a result of various workshops and negotiations, the final PUHTI data consisted of young people’s sports licenses, drug purchases and sales advertisements in the TOR online underground marketplace, and grocery sales. The data were presented according to all 36 postal code areas of Tampere. In addition, data for socioeconomic profiles of the areas was obtained from the population register.

### Negotiations with the data producers and the reporting tool development

**Figure 1:**
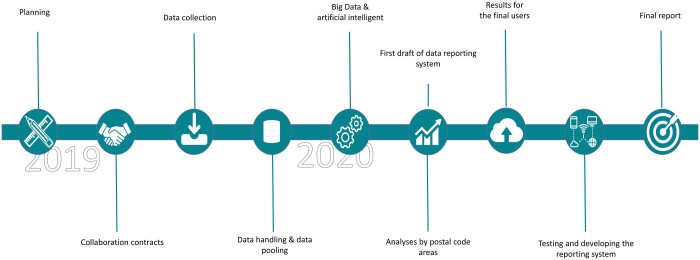
Timetable of the PUHTI pilot study in 2019 and 2020.

Negotiations with the data producers took a lot of time and effort (figure 1), because this type of collaboration was new for them. The main reasons given for providing the data to PUHTI were both to fulfil social responsibility, and being aware that their data were used for the promotion of health and well-being of the people.^20^^,^^25^ Consumers more actively prefer socially responsible companies, hence impacting their profitability.^27^

The contracts between THL and the data producers were signed between June 2019 and October 2020. They precisely defined the terms of data transfer, confidentiality, responsibilities of the parties and ownership and intellectual property rights. The first data sets were received in December 2019. The development of the interactive reporting tool started in January 2020 in collaboration with the health promotion team of Tampere, and it was released in March 2020.

### Feedback from the end-users

The interactive reporting tool was tested for eight months by seven end-users, who were suggested by the main contact person from Tampere city’s health promotion unit, due to their expertise, and possibility to utilize the PUHTI data in their work. The end-users were health and well-being planners and managers from Tampere.

During two commenting rounds, all end-users were interviewed individually in videoconference settings, and written comments were received from three of them. The first interviews were conducted in May 2020, when the reporting tool had been available to users for a couple of months. The second interview round was conducted in November 2020, when the reporting tool had been updated based on feedback from the May 2020 interviews.

### Data collecting and statistical analyses

We aimed at area profiling: data on sports license holders, substance abuse and grocery sales were collected to be presented according to all 36 postal code areas in Tampere. The postal code boundaries and demographic data for area profiling were obtained from Statistic Finland^28^ and from Tampere. PUHTI data were available nearly real time. Data on sport licenses and grocery sales are updated in every 3 months, and data on drug advertisements daily.^25^

*Data on sport license holders* covered sport licenses by sport type, license type (hobby/competition), age (7–17 years), gender and postal code area. It was provided by a Suomisport digital service for sports and physical activity used by over 4000 sports clubs and 710 000 club members in Finland. It provided information on 150 different sport types in 2020, including all the most common sport types, except figure skating, football, basketball, gymnastic and horse riding. Data were supplemented with data from the three associations covering football, gymnastics, and a local multi-sport club. The data from different postal code areas were compared by calculating the number of licenses in the region in proportion to the same-aged population. Aggregated data by postal code areas were sent to THL and analysed using the R-program.

*Data on substance abuse* was collected by studying the amount, drug type, price and postal code area from the drug purchases and sales advertisements in the TOR online underground marketplace for illicit drugs, which in 2020 was the main drug sales channel in Finland. A small amount of alcohol, tobacco and snuff products are also sold/bought there. TOR is freely available and allows anonymity of the user’s location and identity. The market board consists of several sub-boards dedicated to discussions on sales and purchases of drugs. PUHTI analysed the Tampere sub-board. One advertisement corresponded to one potential sale or purchase. The number of sales announcements was seen to describe the supply and availability of drugs, and the purchase announcements the demand and indicated problems in availability. References to drugs were recorded on a weekly basis according to the total number, region and type of notification. The location of the drug seller/buyer was not revealed by the TOR network, but the users mentioned it in most cases in their own advertisements, like ‘cannabis is available/wanted in Area x’. Identical or almost identical advertisements were excluded if they were encountered within 15 min. The data were analysed by a drug data analysis expert, also with the help of artificial intelligence.

**Figure 2:**
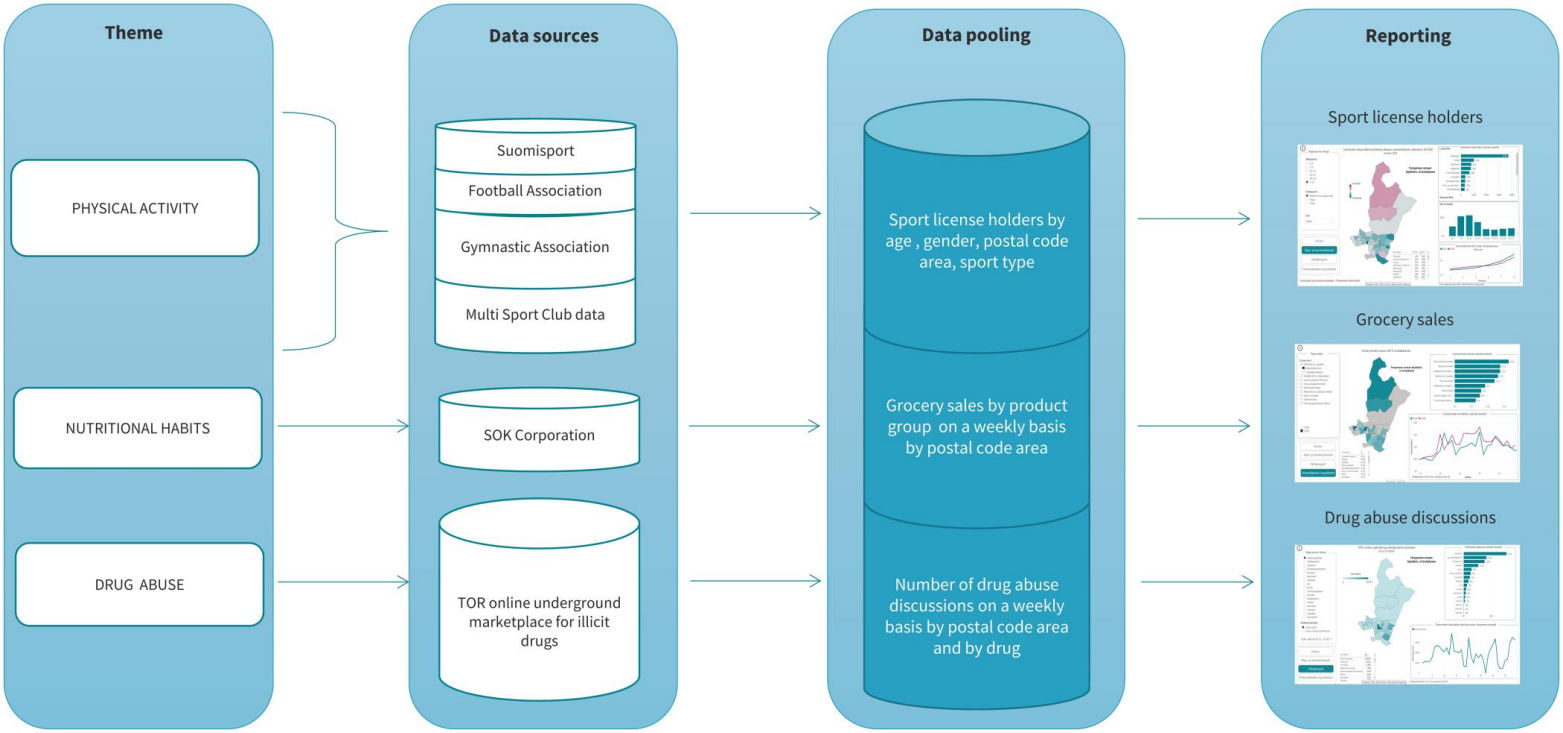
Information flow for data accumulated about the people’s everyday lives—from data mining to the interactive reporting tool. PUHTI pilot study 2019 and 2020.

*Data on grocery sales* were based on grocery store sales provided by the SOK Corporation, which in 2020 was Finland’s largest grocery store chain with a market share of 46%.^29^ The data included daily grocery store sales by store, postal code area and product group (energy drinks, fast food, meat, fish, fruits, vegetables, tobacco and mild alcohol). The product group sales were compared to the total sales in different regions. Data showed how much money was spent on each product group in the region on average from all purchases. The sales of week 9 of 2019 were chosen as the base period, with which the sales of the following weeks were compared. Aggregated data by postal code areas was provided to THL and analysed by using the R-program. The data of all three indicators were pooled into one database. Figure 2 describes the data management process.

### Interactive reporting tool

Creating the interactive reporting tool was the final step in PUHTI. The front page provides (a) summary view of sport license holders, substance abuse and grocery sales data in Tampere, (b) access to the data-specific reports containing the postal code level information and (c) area profiling.

Area profiling was carried out by cluster analysis. This included basic statistical background information: the share of households with children, the share of people with higher education, the share of rental apartments, the unemployment rate and average annual income of citizens over 18 years.^26^ PUHTI information on the proportion of children and young people with sports license holders and the number of drug advertisements were also included in the area profiles. Finally, the areas were divided into four categories:

- Area type 1: More families with children, more sports license holders, high income, highly educated people and more ownership apartments
- Area type 2: More families with children, more sports license holders and high income
- Area type 3: Fewer families with children, fewer sports license holders, more rental apartments and more drug abuse
- Area type 4: Fewer families with children, fewer sports license holders, more rental apartments, more drug abuse, more unemployed people and low incomes

### IT solutions for the database

Data editing and analyzes were carried out with R software version 4.0.2. The GitBucket service was used for code version control. The study’s interactive reporting view was made using Microsoft’s Power BI program, which is a tool for data reporting and analysis. The drug data analysis expert carried out the text analysis of the drug data on the Finnish IT Center for Science’s cPouta cloud server.

### Research ethics and data protection

The study was planned and implemented in accordance with the criteria of good scientific research and using ethically sustainable data acquisition, research and evaluation methods. The data contained no individual-level information or items which would enable indirect identification of any persons. The reporting tool was available only for internal use among seven end-users, and it was not publicly available. A data privacy notice was published on the study, although it did not process any personal data. The data were further aggregated and anonymized when it was handed over to THL to secure the privacy and confidentiality of the people living in the study area.

## Results

### Main results

**Figure 3:**
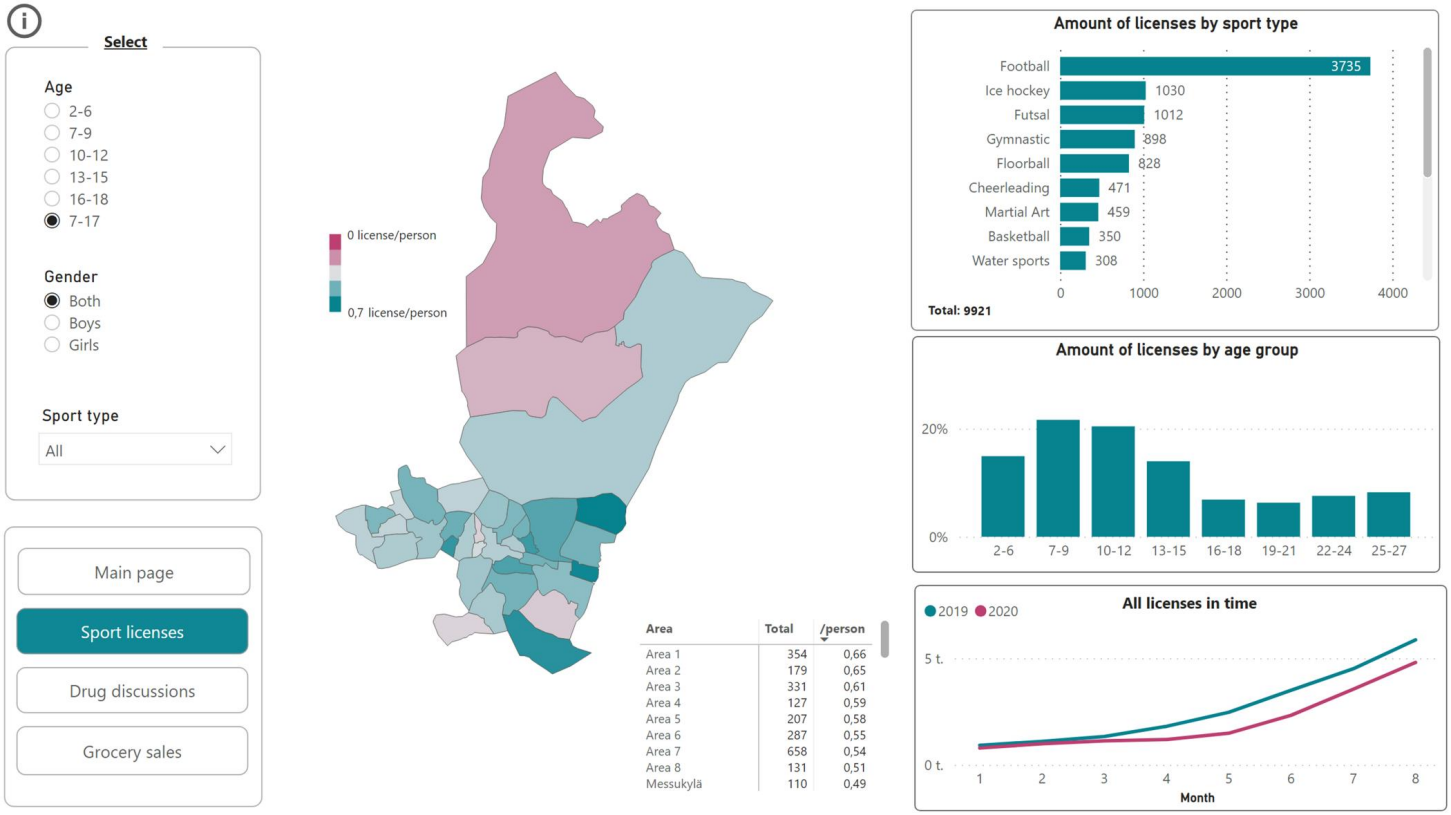
View from the interactive reporting tool: Number of sport license holders in each age group in each postal code area in Tampere: a) total number, b) licenses per inhabitant.

The study showed that buying unhealthy food and alcohol, selling or buying drugs and participating in organized sport activities differed by postal code areas according to its socioeconomic profile in the city of Tampere. There were approximately 0.40 sport licenses per inhabitant with a range by area from 0.20 to 0.66 among 7–17 years old, and football was the most popular sport in all areas in 2020 (figure 3). Cannabis and benzodiazepines were the most purchased and sold drugs, and drug dealing was focused on two postal code areas. Citizens spent an average of 13€ on alcohol and tobacco purchases, with a range by area from 8€ to 40€ out of purchases worth 100€. The end-users found that the new type of data brought added value for the health promotion work at the local level.

### End-user experiences on the PUHTI reporting tool

All end-users felt that the PUHTI data were meaningful for them, and it brought added value for supporting well-being and health promotion work in their city. The interactive reporting tool was perceived as a good tool for planning, managing, allocating resources, applying project fundings and preparing forecasts for the health promotion activities in the city. As PUHTI data are available nearly real time, it provides early signal detection about lifestyles, nutrition, substance abuse and consumption habits. The data raised questions whether the postal code-based differences were due to the socioeconomic factors, or if there was something else behind the issues that should be studied further. The tool helped to open discussions in the multi-sectoral well-being group and fastened its reactions to the emerging health-related challenges with interventions. All the interviewees stated that they would need this type of information systematically in the future.

As parties who could benefit the most from the report, the interviewees mentioned health and well-being planners, planners in other departments of the city and policy makers. They highlighted that the users need to understand the data context to avoid misinterpretation, because there may be several factors that explain the differences between the areas.

Interviewees mentioned several concrete examples on activities in which PUHTI data could or had already been used in Tampere. These included: the development of suburban areas, preventive drug abuse activities at schools when there is a peak in the drug dealing in the area, allocating low-threshold exercise opportunities to the areas and groups needed, planning youth activities, planning meals served at workplaces and school canteens and planning the home economic education at schools. The utilization of the PUHTI data in the municipal welfare report, which is a statutory task for municipalities in Finland, was also seen as important.^30^

## Discussion

The PUHTI study was carried out to explore the possibilities of utilizing the data accumulated about the people’s everyday lives in supporting municipal experts and decision-makers to plan, carry out and lead health promotion activities at the local level. PUHTI revealed differences in both socio-economic profiles and health behaviour according to postal code areas. The end-users, health promotion specialists in Tampere, found that the PUHTI data made the local health inequalities visible, and the local key public health challenges could be identified in greater detail than before. They felt that data helped the managers to allocate resources and health promotion interventions to the areas that needed them most.

The PUHTI showed the similar results as in the previous studies: user-generated big data are seen as a valuable tool for providing new perspectives for health and well-being promotion specialists at the local level. New data sources bring added value because they provide information in nearly real time with low costs from a larger number of people, and potentially from more diverse demographic groups, than the traditional health data. New data sources may give important information also from those who are difficult to reach by the traditional ways: for example, on those citizens not using health services or not participating in health surveys.^5^^,^^7^^,^^10^^,^^16–18^^,^^21^^,^^23^^,^^31^^,^^32^ The ability to access and combine diverse health related data can provide early signal detection long before there is a need for health services, enrich analysis and evaluation and help decision-making.^5^^,^^10^^,^^23^^,^^31^ The data accumulated about people’s everyday lives outside the health service system can provide a practical tool for knowledge-informed management at the local level, compliment traditional information and renew perspectives. However, according to our experiences, the maintenance of this type of system requires sustainable funding from the participating municipalities and regions.

### Strengths and weaknesses

We recognized the following strengths and weaknesses in our study:

1. Sampling: Sample is never totally representative, but relatively much is known about the residents’ and their socio-demographic background in our study, because Finland has comprehensive and up-to-date population registers in national and municipal use. In many other countries, such information may not be so comprehensively available.
2. Qualitative reliability: The large size of the data may not necessarily mean the information provided is more accurate and valid. It may lead to a misconception about the reliability and the statistical significance of the results: for example, everyone does not use TOR network to buy/sell drugs, all citizens do not buy food from their own postal code area and register on sport license holders does not cover all large sports groups.^10^^,^^33^
3. Data on substance abuse: It has been studied that TOR network sales and purchase notifications supported and complemented the traditional data sources (wastewater-based drug monitoring and drug crimes reported to the police) in drug abuse. All three data sources gave a unified picture from the different perspectives of the changes in drug situation in time in Tampere.^34^ The location of the drug seller/buyer was not revealed by the TOR network, but the users mentioned it in most cases in their own advertisements, such as ‘cannabis is available/needed in Area x’, which increased the validity.
4. Data on sports license holders: Data included 150 different sport types in 2020, and it covered all the most common sport types, except figure skating, football, basketball, gymnastic and horse riding. Data were supplemented with data from the three associations covering football, gymnastics and a local multi-sport club. In the end, data was missing for the most common sport hobbies only for figure skating and horse riding. Suomisport database included all 7 to 17 years old participating in competitive activities in member sports associations, because the sport license in required in the competitive sport activities in Finland. In several sports also a hobby license is required, so the database includes these as well.
5. Data on grocery sales: The customer data describes only partially individual or regional consumption as it does not directly indicate who has consumed the products. For example, people may offer their shopping bag products to guests and pets^18^ or make the purchases for other people living outside the area. The customers might use certain grocery stores to get specific products and the data may be limited to big shops. For example, the vegetables may be cheaper somewhere else, or a customers can buy products from a specialized store in the neighbouring area or from the store near their workplace. In some areas have large grocery stores, where the shopping baskets may be more versatile due to the wider range of products.^21^ In PUHTI, the grocery sales data were provided by the postal code areas, but purchases could not be targeted by the customer’s postal code area. In the next stage of PUHTI, the distribution of grocery purchases is better targeted by utilizing information from grocery stores about the customers’ residential locations. Additionally, alcoholic beverages with 5.5% alcohol or higher by volume have to be bought from the specific alcohol stores in Finland,^35^ so PUHTI data did not cover those. The data did not either cover all tobacco products, because the electronic cigarettes and snuff products are not sold in the grocery stores.

Despite the attractiveness and promising potential of utilizing new types of data, critical evaluation on its accuracy, quality, reliability, validity and meaningfulness is important before drawing inferences from the results and providing them to stakeholders. It is crucial to reflect the data in the local multidisciplinary teams that knows the local context. The reflections are needed to avoid strengthening the existing health inequalities and making wrong interpretations.^10^^,^^21^^,^^23^^,^^24^^,^^32^^,^^33^^,^^36^

#### The future of the PUHTI study

The PUHTI pilot study was so successful that three new municipalities and one well-being services county wanted to join the PUHTI follow-up study (2023–2025). The goals of the follow-up are largely the same as in the pilot study, but the database will be expanded, and more themes will be explored. These include: the social exclusion of young people, exercise and hobbies, healthy diets and regional differences. The reporting view will be developed and available for wider use, and the use of information in health and well-being promotion will be implemented more strongly. The data pool is intended to also serve at the national (e.g. various ministries) and regional level as well as in the largest cities.

#### Future needs

There is no doubt that the traditional health data collection methods—administrative registers and health surveys—will be the cornerstone in the database for health promotion also in the future. However, in order not to be constantly a step or two behind the health and social challenges in the area, municipalities and regions need multiple data sources to make better strategies to improve the health and well-being of all citizens. A single data source seldom provides solutions but combining multiple relevant data sources opens a completely new perspective for health promotion at local and regional levels. An analysis that also includes new types of data sources may challenge prevailing perceptions and offer new solutions to important problems—such as reducing health inequality. Ideally, all sectors of society should work together to promote holistic well-being for the citizens.

## Data Availability

Data from the PUHTI pilot study are not publicly available due to its sensitive nature. It is only intended for the use of the end users of the city of Tampere, and the PUHTI study workers at the THL. Key points Administrative registers and health surveys are the cornerstone in the management of health and well-being at the local level. Data accumulated about the people’s everyday lives outside the health service system—owned for example by local companies and non-governmental organizations—can provide additional information on the people’s health behaviour at the local level, also by population groups. Combining new data sources with the traditional health data sources open a completely new perspective for health promotion work at the local and regional level. New data sources bring added value because they provide information in near real time with low costs from a larger number of people and potentially from more diverse demographic groups than the traditional health data, but the new data sources need to be evaluated to consider their limitations.
